# Perspectives on label-free microscopy of heterogeneous and dynamic biological systems

**DOI:** 10.1117/1.JBO.29.S2.S22702

**Published:** 2024-02-29

**Authors:** Dan L. Pham, Amani A. Gillette, Jeremiah Riendeau, Kasia Wiech, Emmanuel Contreras Guzman, Rupsa Datta, Melissa C. Skala

**Affiliations:** a University of Wisconsin—Madison, Department of Biomedical Engineering, Madison, Wisconsin, United States; b Morgridge Institute for Research, Madison, Wisconsin, United States

**Keywords:** label-free, microscopy, heterogeneity, open-source software, artificial intelligence, cell dynamics

## Abstract

**Significance:**

Advancements in label-free microscopy could provide real-time, non-invasive imaging with unique sources of contrast and automated standardized analysis to characterize heterogeneous and dynamic biological processes. These tools would overcome challenges with widely used methods that are destructive (e.g., histology, flow cytometry) or lack cellular resolution (e.g., plate-based assays, whole animal bioluminescence imaging).

**Aim:**

This perspective aims to (1) justify the need for label-free microscopy to track heterogeneous cellular functions over time and space within unperturbed systems and (2) recommend improvements regarding instrumentation, image analysis, and image interpretation to address these needs.

**Approach:**

Three key research areas (cancer research, autoimmune disease, and tissue and cell engineering) are considered to support the need for label-free microscopy to characterize heterogeneity and dynamics within biological systems. Based on the strengths (e.g., multiple sources of molecular contrast, non-invasive monitoring) and weaknesses (e.g., imaging depth, image interpretation) of several label-free microscopy modalities, improvements for future imaging systems are recommended.

**Conclusion:**

Improvements in instrumentation including strategies that increase resolution and imaging speed, standardization and centralization of image analysis tools, and robust data validation and interpretation will expand the applications of label-free microscopy to study heterogeneous and dynamic biological systems.

## 1 Introduction

**Fig. 1:**
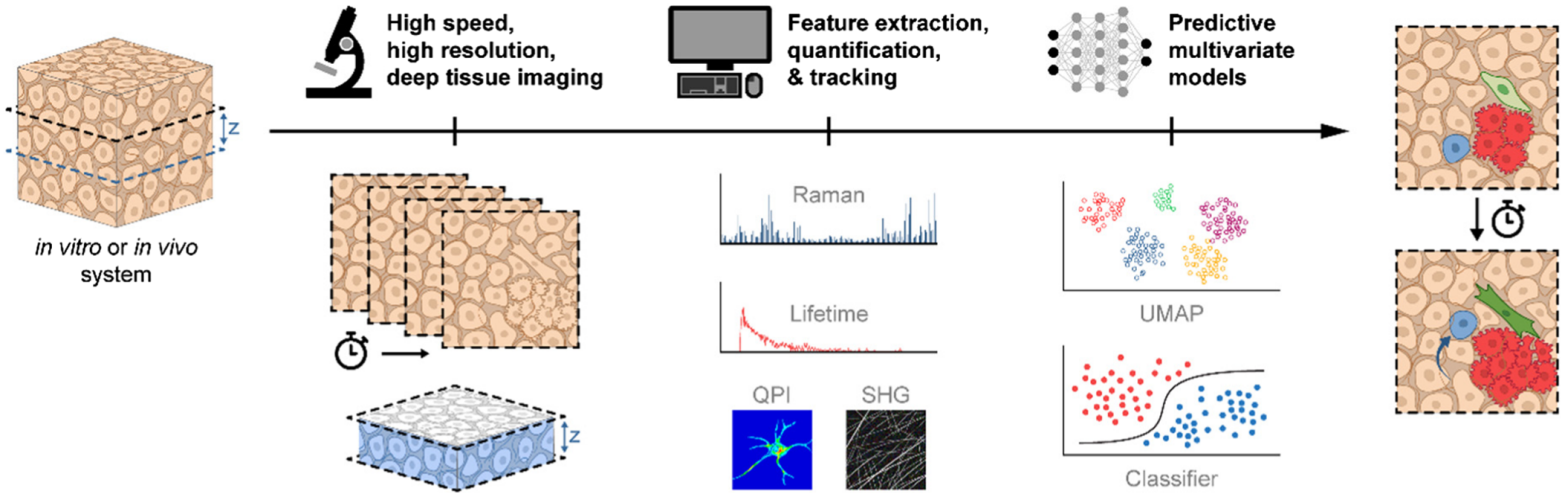
Future vision for label-free microscopy of heterogeneous and dynamic biological systems. 3D image stacks could be non-invasively generated deep within *in vitro* and *in vivo* systems at high-speed and resolution. Several label-free sources of contrast could be extracted from Raman spectra, fluorescence lifetimes, and phase shifts to define cell phenotypes (e.g., immune cell activation, cancer cell growth, stem cell differentiation) and behaviors (e.g., cell migration). Extracellular features including collagen content and morphology can also be visualized using label-free methods, such as second harmonic generation microscopy. Multivariate models including data visualization [e.g., uniform manifold approximation and projection (UMAP)] and predictive artificial intelligence (AI) models can be built from label-free sources of contrast to determine real-time function or predict future behavior within intact samples. Rightmost images depict dynamic changes in cell function captured with a label-free microscopy time series, including fibroblast activation (green), immune cell migration (blue), and cancer cell proliferation (red) within a heterogeneous tissue environment. Overall, this framework will provide single-cell information on molecular, functional, and structural features that will enable critical insights into dynamic, heterogeneous, living samples over multiple timescales.

Sophisticated *in vivo* and *in vitro* models have been developed to study normal physiology, disease development, and to test novel treatments. These biological systems are heterogeneous and dynamic, comprising various cell types with specialized functions and complex spatiotemporal interactions. However, standard techniques (e.g., histology, flow cytometry, plate-based assays, whole animal bioluminescence imaging) to assess these biological systems are time consuming, invasive, and/or lack the ability to characterize heterogeneous and dynamic biological processes in the native context. Non-destructive, label-free microscopy techniques can bridge these gaps to study biological models encompassing *in vitro* two-dimensional (2D) and three-dimensional (3D) cell culture, primary human samples (e.g., peripheral blood, tumor resections) or *in vivo* animal models (e.g., mouse, zebrafish) in static (single time point) or dynamic (time course) systems. Label-free microscopy techniques have the potential to non-invasively acquire 3D image stacks from deep within *in vitro* and *in vivo* systems at high-speed and resolution to capture molecular and morphological features of single cells. In addition, numerous sources of label-free molecular contrast including Raman spectra, autofluorescence lifetimes, and spectral properties provide multivariate measurements of cell function that can offer unique insights.^1^^,^^2^ However, improvements in instrumentation, image analysis, and image interpretation are necessary to fully realize the capabilities of label-free microscopy (Fig. 1).

## 2 Rationale for Label-Free Microscopy to Monitor Heterogeneous Cell Function Over Spatiotemporal Dynamics

Characterization of heterogeneity and dynamics within biological models is crucial to understand the complex interaction among several cell types and extracellular components over space and time. However, current bioassays commonly used in biomedical research face several challenges in assessing dynamic and heterogeneous living systems. Many standard assays, including ELISA, plate-based assays, extracellular flux analysis, and whole animal bioluminescence imaging, provide bulk population measurements of intra- and extra-cellular metabolites or cytokines, or the functional state of the overall system. These assays are useful for studying homogeneous cell populations. However, when more than one cell type is present within the population, it is difficult to attribute the changes observed in bulk measurements to functional changes in a specific cell type. Meanwhile, single-cell assays for heterogeneity assessment, such as histology, flow cytometry, and single-cell RNA sequencing, often require intensive and destructive sample preparations that are not suitable for continuous assessment of dynamic cellular processes. In addition, despite its ability to capture heterogeneous cell populations, microscopy with labeled systems (e.g., transgenic reporter lines, fluorescent contrast agents) can alter the native biological context, while facing challenges with specific expression and/or delivery of labeling molecules. Photobleaching is another important limitation in labeled microscopy, where the loss of labeling signals prevents tracking of cells or subcellular features over time. Here, we discuss in detail the heterogeneous and dynamic nature of three example research areas (cancer, autoimmune disease, and tissue and cell engineering) to justify the applications of label-free microscopy in these areas.

### 2.1 Cancer Research

Tumorigenesis and treatment response are affected by the complex tumor microenvironment (TME), which is comprised of multiple cell types (such as immune, stromal, and tumor cells) and non-cellular components [such as the extracellular matrix (ECM) and exosomes].^3^^,^^4^ Within these cellular and noncellular components, there are several subpopulations with heterogeneous functions that actively influence the TME.^4^ For example, tumor-infiltrating immune cells include both anti-tumor cells (such as cytotoxic T cells and M1 macrophages) and pro-tumor cells (such as regulatory T cells and M2 macrophages).^5^ Meanwhile, growth factors secreted by tumor cells support stromal cell viability and ECM stiffening, which in turn facilitate tumor cell proliferation and metastasis while creating a physical barrier for immune cell infiltration.^6^ Dynamic interactions among these cellular and noncellular components within the TME directly affect immune functions, ECM remodeling, and drug sensitivity, ultimately determining patient outcomes.^3^ In addition, the TME can alter oxygen and nutrient gradients, further contributing to inter- and intra-patient heterogeneity in cancer cell drug response,^7^ while also increasing the diversity within and between cell types *in vivo.*^8^ Therefore, multiple *in vitro* and *in vivo* systems have been developed to model these characteristics during tumorigenesis, tumor progression, and treatment response, including patient-derived organoids, *ex vivo* primary tumor slices, and xenograft animal models.^9^ It is important to recognize that both cancer development and treatment response are continuous processes rather than discrete, static events. While destructive techniques, such as histology, flow cytometry, and single-cell RNA/DNA sequencing, provide important insights into the TME, these techniques are limited to snapshots in time. Hence, they do not fully capture the dynamic and heterogeneous characteristics of cancer. Non-invasive label-free microscopy can be used to monitor heterogeneous functions and quantify dynamic changes in these *in vitro* and *in vivo* tumor models. This enables a better understanding of the complex cancer biology in its native context and supports the development of novel cancer treatments.

### 2.2 Autoimmune Diseases

The heterogeneity of the immune system—reflected in the diversity of the T cell receptor repertoire and $>10^{12}$ distinct antibodies secreted by B cells—creates a substantial challenge to study autoimmune diseases.^10^^,^^11^ Common autoimmune disorders, such as systemic lupus erythematosus (SLE) and inflammatory bowel disease (IBD), are associated with metabolic abnormalities, improper cell–cell interactions, and atypical cell subpopulation ratios.^12^^–^^14^ For example, a high Th17/Treg (regulatory T cell) ratio shows a positive correlation to disease activity in SLE patients.^15^ Single-cell resolution is therefore crucial to evaluate these characteristics within autoimmune disease models. Due to its ability to characterize heterogeneous cell populations in dynamic environments, label-free microscopy is an attractive method for studying autoimmune diseases. In addition, label-free sources of contrast are often continuous variables, which can capture the known spectrum of dynamic activity within immune cells.^16^^–^^19^

In addition to providing a better understanding of autoimmune disease, label-free microscopy has potential clinical applications. Current methods for diagnosing SLE are time-consuming and often require months or years for a proper diagnosis due to non-specific and varied symptoms manifested in patients.^20^^,^^21^ Label-free microscopy offers unique sources of contrast based on endogenous cellular features. This can increase the sensitivity and specificity to screen for abnormalities in cellular functions and cell–cell interactions associated with autoimmune diseases. Label-free techniques also alleviate sample preparation, hence, reducing sample characterization time in clinics. Because of these features, label-free microscopy holds great promise for clinical translation. Furthermore, recent research suggests that targeting immune cell metabolism could be an effective treatment in SLE and IBD.^22^^,^^23^ Label-free microscopy modalities that are sensitive to cell metabolism could improve drug screening and support the generation of multimodal diagnosis, monitoring, and treatment tools for autoimmune disorders.

### 2.3 Tissue and Cell Engineering

Engineered tissues and cells have been used as therapeutic products, disease models, and treatment screening platforms. However, variability within and among batches remains a major challenge for tissue and cell engineering.^24^ For example, brain organoids generated from induced pluripotent stem cells display varying differentiation potential and maturation rate, which limits their use in neuronal maturation studies.^25^ Similarly, the manufacturing of engineered immune cells, such as chimeric antigen receptor (CAR) T cells, faces variability in transgene incorporation efficiency and cell behavior, which leads to inconsistent responses among patients.^26^

In a closed-loop manufacturing workflow, analytical methods that are destructive or rely on labels often require removing samples from the in-process products. Removing cells from a culture for analytical testing is common in CAR T cell development and manufacturing, although this approach risks product contamination and destroys the sampled cells. However, cells cannot be removed during tissue manufacturing because intact structure is required for functional tissues. Hence, removing cells during the tissue manufacturing process results in product loss. Label-free microscopy can non-invasively monitor heterogeneous and dynamic cell functions within and among cultures without sample destruction while enabling timely interventions to ensure consistent and potent products. Molecular or structural features extracted from label-free microscopy can also serve as critical quality attributes for engineered tissues and cells. Importantly, the non-destructive nature of label-free microscopy facilitates expansion of rare cell types such as tumor-infiltrating lymphocytes or the exclusion of contaminants such as pluripotent stem cells in engineered cell and tissue products.^27^^–^^29^ Overall, label-free microscopy is advantageous for tissue and cell engineering as it allows non-invasive identification and continuous monitoring of single cells to improve product quality.

## 3 Current Landscape of Label-Free Microscopy

### 3.1 Hardware

Numerous label-free microscopy techniques exist to visualize cell morphology, migration, molecular features, and function. Cell morphology and migration can be monitored with quantitative phase imaging (QPI) methods pioneered by Prof. Popescu that rely on differences in index of refraction for contrast,^30^ as well as computational microscopy,^31^^,^^32^ scattered light microscopy,^33^ and traditional brightfield or differential interference contrast microscopy.^34^ Similarly, optical coherence tomography (OCT) generates high-resolution images of tissue structure to assess thickness, density, and organization of cells and the ECM.^35^ These modalities are advantageous for rapid, low-cost imaging and simplified optical design to track dynamic cell movements and interactions, but often lack molecular contrast to identify specific cellular and subcellular populations. To identify these subsets, label-free molecular microscopy has been developed to monitor intrinsic sources of biochemical contrast in cells, including Raman microscopy,^36^ spectral imaging,^2^ and nonlinear microscopy.^37^ Raman microscopy measures the vibrational modes of molecular bonds to identify the presence and abundance of specific biomolecules, such as proteins, lipids, and nucleic acids. Nonlinear microscopy can separate endogenous molecular sources of fluorescence (e.g., metabolic cofactors, retinoids) with spectral detection or fluorescence lifetime imaging microscopy (FLIM).^38^ Meanwhile, second harmonic generation (SHG) microscopy specifically highlights the distribution of collagen and other non-centrosymmetric molecules within the tissue.^39^ These approaches provide additional insights into molecular and functional behaviors in cells and their environment.

**Fig. 2:**
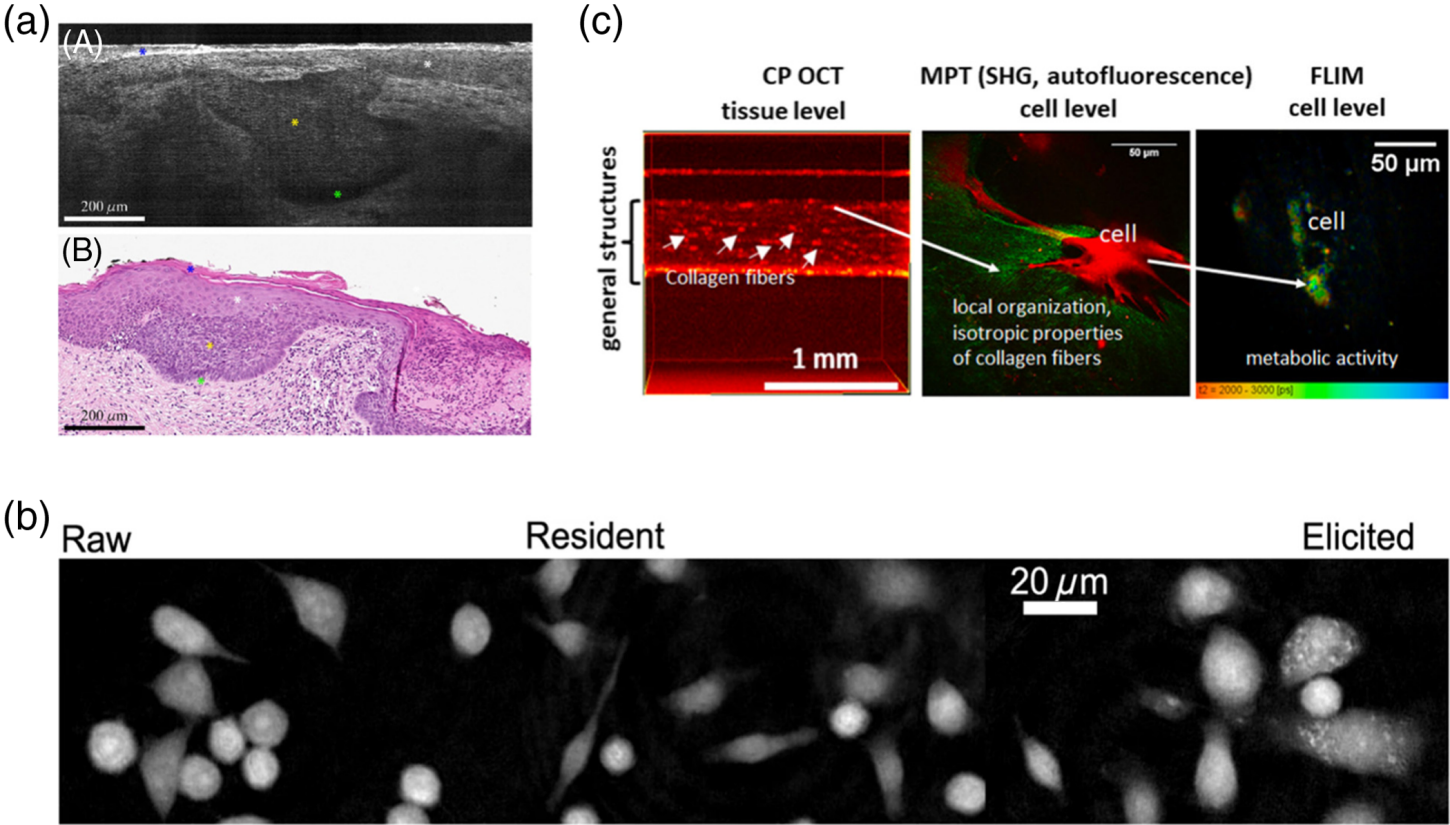
Applications of label-free microscopy in (a) cancer research, (b) autoimmune disease research, and in (c) cell and tissue engineering. (a) Line-field confocal OCT image (top) and corresponding histopathological examination (bottom) of a superficial basal cell carcinoma. Blue star: stratum corneum; white star: epidermis; yellow star: clusters of tumor cells; green star: cleft between tumor cell islands and dermis. Adapted with permission from Ref. 44. OCT was acquired with a supercontinuum laser at 800 nm center wavelength and 250 nm full width half maximum. (b) Raman spectral properties and 301 morphological features extracted from QPI classified different macrophage populations and their activation states, which are implicated in autoimmune diseases, with up to 97% sensitivity. Adapted with permission from Ref. 45. 780 nm laser diode was used for QPI light source. Raman spectroscopy was performed with 532 nm laser excitation. (c) CP OCT and multiphoton tomography (MPT) based on SHG and autofluorescence FLIM of NAD(P)H showed the formation of collagen fibers and increased oxidative metabolism in dermal papilla and fibroblast cells over 14 days of engineered skin tissue (dermal equivalent) development. MPT image shows interaction of collagen (green) and individual cells (red). Adapted with permission from Ref. 46. CP OCT was performed with a 1300 nm center wavelength source. 740 nm excitation wavelength was used for both SHG and FLIM of NAD(P)H, with detection range of 373 to 387 nm and 410 to 650 nm, respectively.

Label-free microscopy can characterize cellular heterogeneity and is amenable to kinetic measurements over space and time within unperturbed systems. These features are especially attractive for tracking important subsets of cells in living systems, supporting applications of label-free microscopy for cancer, autoimmune disease, and tissue and cell engineering. For example, high-speed live cell interferometry, a QPI methodology, has been used to monitor single cell biomass changes with treatment in mouse breast tumor xenografts,^40^ and to quantify biomass in human breast cancer organoid models.^41^ Autofluorescence FLIM and SHG have highlighted the interactions between immune, tumor, and stromal cells in mouse melanoma *in vivo*^42^ and revealed the role of fibroblasts in ECM remodeling during cancer cell metastasis.^43^ Line-field confocal OCT has also been introduced into the clinic for label-free assessment of skin lesions to identify cancerous characteristics and monitor healing post-treatment with an isotropic spatial resolution down to 1  μm, acquisition time of 10  frames/s, and imaging depth of 0.5 mm [Fig 2(a)].^44^ Meanwhile, as macrophage dysfunction is implicated in the pathogenesis of several autoimmune diseases including SLE and type 1 diabetes, QPI and Raman scattering spectroscopy have been used to characterize morphological and molecular features of macrophages from multiple sources (cell line versus resident and elicited peritoneal macrophages).^45^^,^^47^ These label-free measurements (including Raman spectral properties and 301 morphology features representing size, shape, intensity, radial distribution, and texture extracted from QPI images) classified macrophages and their activation states with up to 97% sensitivity and specificity [Fig. 2(b)].^45^ Hence, label-free methods could be used to characterize autoimmune diseases and understand autoimmune flare-ups.^45^^,^^48^^,^^49^ Applications of label-free microscopy in tissue and cell engineering have also been demonstrated, as autofluorescent lifetimes and intensity of metabolic coenzymes NAD(P)H and FAD collected with two-photon FLIM are sensitive to heterogeneous T cell function and predict differentiation efficiency for cardiomyocytes derived from induced pluripotent stem cells.^50^^–^^52^ Finally, multiple modalities can be combined to assess engineered skin tissue [Fig. 2(c)],^46^ where the advantages of larger field of view (4×4  mm) and imaging depth (1.7 mm) offered by cross-polarization (CP) OCT complement the high resolution and single cell information from multiphoton SHG and FLIM to characterize tissue structure (collagen organization) and function (cell metabolism) across different scales.

However, current label-free microscopy systems are still limited in spatial resolution, speed, and imaging depth. While cellular and subcellular features are important to capture population heterogeneity, few label-free microscopy systems offer sufficient resolution to visualize cellular organelles, cell-cell synapses, and cell–ECM interactions. Meanwhile, label-free modalities with high resolution and molecular contrast, such as FLIM and Raman microscopy, often rely on laser scanning and require either high photon counts for reliable fluorescent lifetime fitting or long exposure times to build up thousands to millions of pixel-wise Raman spectra.^53^^,^^54^ For instance, laser-scanning autofluorescence FLIM of the metabolic coenzyme NAD(P)H requires long integration times (on the order of tens of seconds) per field of view due to the low quantum yield of NAD(P)H (∼2% for NAD(P)H, compared to ∼80% for the exogenous fluorophore fluorescein).^55^^–^^58^ This increases image acquisition time, especially for laser-scanning techniques, limiting both the imaging throughput and the capacity to capture fast biological processes such as cardiomyocyte contraction or immune cell movement.

Limited imaging depth also poses a challenge for the adoption of label-free microscopy, especially for non-transparent *in vivo* models such as mice. The attenuation of light in tissue is represented with $\mu_{t}$, the optical attenuation coefficient.^35^ While $\mu_{t}$ is dependent on the wavelength and tissue type, it typically ranges between 1 and $50\;{\mathrm{cm}}^{-1}$ for common tissue types (such as skin and fat) and can reach up to $1000\;{\mathrm{cm}}^{-1}$ for blood.^59^ The attenuation coefficient decreases in the near infrared and infrared windows, hence longer excitation wavelengths allow for greater penetration depth; however, the imaging depth is still limited to the millimeter scale from the tissue surface.^60^^,^^61^ For example, OCT in the near-infrared window provides millimeter scale penetration depths into scattering tissues and has been used in several *in vivo* models for label-free imaging.^44^^,^^46^ Meanwhile, the inherently low signal-to-noise ratio (SNR) of intrinsic sources of contrast compared to engineered contrast agents further exacerbates signal attenuation with deep tissue imaging.^62^ Therefore, the use of label-free microscopy for non-invasive monitoring of unperturbed systems *in vivo* remains a challenge.

### 3.2 Image Analysis

Standard analysis workflows for label-free microscopy include several steps: (1) segmentation and tracking to identify and monitor objects, (2) feature extraction from those objects to yield quantitative measurements, and finally (3) metadata handling, such as visualization, pattern characterization, and generation of predictive multivariate models. With the integration of neural networks and deep learning, existing tools have achieved fast and accurate automatic segmentation of label-free images, including segmentation of cells (CellProfiler,^63^ cellpose,^64^ and StarDist^65^), collagen (CT-Fire^66^ and CURVEAlign^67^), and mitochondria (MiNA,^68^ Mito Hacker,^69^ and U-Net^70^), as well as object tracking (btrack,^71^ TrackMate,^72^ and Trackpy^73^). While the segmentation tools can characterize intra- and intercellular heterogeneity, tracking tools can monitor dynamic processes. Segmentation and tracking tools can be used to extract numerous features from each object, such as mitochondrial morphology and network structure; collagen fiber alignment and density; cell size, shape, and co-localization; and cell and/or organelle speed and direction.

Supervised machine learning models are under development to visualize, identify, and correlate patterns in multivariate label-free images for medical diagnostics and biomanufacturing. For example, machine learning classifiers have been used with QPI for automatic Gleason grading of human prostate cancer specimens^74^ and with FLIM to automatically assess maturation of engineered cartilage.^75^ Meanwhile, non-supervised models are used to identify patterns within label-free images, including dimensionality reduction and data visualization algorithms, such as uniform manifold approximation and projection (UMAP),^76^ t-distributed stochastic neighbor embedding (t-SNE),^77^ and principal component analysis (PCA).^78^ For example, UMAP has been used to visualize clustering of single-cell RNA expression and Raman spectral profiles.^79^ Similarly, PCA of QPI features have identified biomarkers for drug screening in breast cancer.^80^ Examples of image analysis tasks, computational tools, and their application to label-free microscopy studies are provided in Table 1.

**Table 1:** Examples of current tools available for label-free image analysis and their applications.

Tasks	Tools	Example applications in label-free microscopy
Automated image segmentation and feature extraction	Single cell segmentation: CellProfiler, cellpose, StarDist	Segmentation and quantification of autofluorescence signals from individual cell cytoplasms to identify changes in T cell metabolism upon activation^51^
	Collagen segmentation and morphology/network analysis: CT-Fire, CURVEAlign	Characterization of collagen fiber morphology and organization from SHG images of colon cancer mucosa versus healthy tissue^81^
	Mitochondria segmentation: MiNA, Mito Hacker, U-Net	Segmentation and characterization of mitochondrial network, morphology, and dynamics (cleavage) following FCCP treatment with high resolution phase microscope^82^
Object tracking	Btrack, TrackMate, Trackpy	Single-cell tracking in label-free brightfield images of pluripotent stem cells during differentiation into definitive endoderm^83^
Multivariate analysis	Dimension reduction and data visualization: UMAP, t-SNE, PCA	Clustering of single-cell RNA sequencing and Raman spectral properties during stem cell reprogramming^79^
	Correlations and classification: machine learning, neural networks	Automated Gleason grading of human prostate cancer specimens based on QPI parameters of tissue biopsies with up to 82% accuracy^74^

While tools to address individual steps within the label-free microscopy analysis workflow exist, they are currently modular and not fully integrated with each other. This integration is especially needed for multi-modality imaging with several label-free microscopy techniques. In addition, there is a lack of centralized and standardized sources for label-free image analysis codes, as current tools were developed by individual labs or organizations that require different programming languages, such as Python or MATLAB. Therefore, these tools often demand a certain level of programming knowledge or other specialized skills to execute and troubleshoot, hindering the translation and adoption of label-free microscopy.

### 3.3 Image Interpretation

Image interpretation remains a challenge for label-free microscopy in biomedical research. For example, in autofluorescence imaging, several fluorescent species with different biological functions can have overlapping spectral properties, which confounds the process of image analysis and data interpretation. More than 14 endogenous fluorophores have been identified in cells that contribute to tissue autofluorescence signals, including structural proteins, such as elastin and collagen, vitamins such as vitamin A and B6, neurotransmitters, lipids, and metabolic coenzymes.^38^^,^^84^ The majority of these autofluorescence biomolecules are excited in the UV range.^38^^,^^84^ This is especially problematic for *in vivo* systems, where it is difficult to isolate the signal of interest and minimize the sources of background signal bleed-through. Meanwhile, the changes in the autofluorescence lifetime, for example, in NAD(P)H, can be due to numerous factors, including protein binding activity, preferred binding partners, and the presence of quenchers such as pH and oxygen.^85^^,^^86^ Similarly, changes in Raman spectra can be subtle, and variability in instrument, sample, and computational processing methods limits consistency between studies and interpretation of underlying biological processes.^87^

Therefore, label-free measurements should be benchmarked with standard assays to accurately interpret underlying biological phenomena. Metabolic subsets of cells identified non-invasively with autofluorescence FLIM are currently validated with metabolic flux analysis, metabolomics, and/or metabolite measurements in media.^17^^,^^51^^,^^88^ Similarly, Raman spectral features can be supported with matrix-assisted laser desorption/ionization mass spectrometric imaging. Single-cell identity assessed with label-free microscopy can also be benchmarked against flow cytometry analysis of intracellular and surface protein markers. However, these assays are destructive to the samples and can only be performed in parallel or at experimental endpoints, which does not fully capture the dynamics obtained with label-free microscopy.

## 4 Strategies to Improve Label-Free Microscopy

### 4.1 Hardware

As technologies advance, label-free microscopy will see improvements in imaging resolution, speed, depth, and molecular specificity to capture greater heterogeneity and faster dynamics in biological samples. For example, single photon avalanche diode (SPAD) arrays are becoming prevalent in microscopy due to their high sensitivity and temporal resolution. As SPAD array technology advances, SPADs could be integrated into label-free microscopy systems, such as light-sheet autofluorescence,^89^ light-sheet hyperspectral Raman,^90^ and QPI light-sheet^91^ to enable high-speed, volumetric images. For example, an SPAD array (192×128  pixels, 1.75  mm×2.35  mm sensor size with dedicated time-to-digital electronics for each pixel) was integrated into a light-sheet geometry, resulting in a 6- to 30-fold decrease in acquisition time per frame for autofluorescence FLIM compared to laser-scanning two-photon autofluorescence FLIM.^89^ Similarly, light-sheet Raman micro-spectroscopy acquired hyperspectral Raman images with a fivefold increase in acquisition speed compared to a confocal Raman microscope.^90^

Alternatively, by coupling label-free techniques with adaptive optics, greater depths and aberration-free image resolution can be attained.^92^ In addition, robust biological interpretation with a high level of confidence can be achieved by combining unique sources of contrast from multiple label-free modalities for correlative studies. For example, QPI and quantitative intensity imaging have been combined to create fluorescence self-interference (SELFI) for super resolution imaging beyond diffraction limit with ˜23 to 50 nm axial resolution up to a few tens of microns depth.^93^ With these advancements in instrumentation, future label-free microscopy systems could achieve high-speed, high-resolution, and volumetric characterization of important cell subsets engaged in fast dynamics, such as beating cardiomyocytes or the formation of immune cell synapses *in vitro* and *in vivo*.

Improvements in the imaging depth, resolution, and speed of label-free microscopy can also be achieved with artificial intelligence (AI) techniques. For instance, developments in neural networks and machine learning could enhance photon-deprived signals from deep sections within 3D samples. In fact, machine learning has recently been used to process label-free FLIM images acquired in low SNR conditions.^94^^,^^95^ This allows reliable recovery of FLIM decays with low photon counts, hence enabling fast image acquisition in deep tissue. For example, lifetime estimates with high accuracy were achieved with 50 times fewer photons per pixel (i.e., 10  photons/pixel for exogenous fluorescence and 30 to 40  photons/pixel for autofluorescence from live cells) compared to ground-truth.^94^ Similarly, an increase in imaging depth has been achieved using deep learning algorithms that combined confocal microscopy and QPI for phase retrieval and tomographic reconstruction.^96^^,^^97^ AI-assisted adaptive optics methods have also been used with nonlinear label-free microscopy to improve imaging depths for high-resolution images.^98^ Meanwhile, deep neural networks have been used to obtain 3D volumetric images from 2D fluorescence images of dyes, such as FITC and Texas Red, to study neuronal activity.^99^ These approaches could be applied to label-free images to achieve greater imaging depths and resolution. These studies collectively highlight the potential of AI techniques to overcome current limitations and enable new opportunities for label-free microscopy, especially to study heterogeneous and dynamic systems in cancer, autoimmune disease, and tissue and cell engineering.

### 4.2 Image Analysis

Improvements in image resolution, speed, and depth will increase the amount of information collected per experiment. Therefore, faster and more accurate segmentation and tracking tools are needed to handle greater numbers of objects and longer time-lapse imaging. The scalability and robustness of these tools will need to be tested with large numbers of annotated images that capture heterogeneous and dynamic information.

Since image analysis is a multistep process, the development of a complete, standardized workflow that integrates current stand-alone modular tools with a user-friendly interface will greatly improve adoption of label-free microscopy. With continuous development, community engagement, and adoption of novel AI tools, centralized libraries will emerge that encompass a range of algorithms and packages to improve the access, scale, and specificity of label-free microscopy. Integrative systems, such as Napari,^100^ ImageJ,^101^ OMERO,^102^ or BioImage Informatics Index,^103^ have started to address these challenges by incorporating plug-ins or tools for specialized image analysis tasks. The whole label-free microscopy community must work together to further expand these centralized tools and develop a standardized analysis workflow from segmentation to feature extraction and metadata modeling. This will streamline the analysis process and increase adoption of label-free microscopy for biomedical researchers, while also improving data integrity and reproducibility for the whole field.

### 4.3 Image Interpretation

Advancements in label-free instrumentation and image analysis will support the validation and interpretation of label-free images. Ongoing developments in multimodal imaging will generate co-registered images between label-free methods and standard assays to further address challenges in image interpretation. AI-assisted image analysis workflows are also critical to extract important features from label-free images and correlate with standard biomarkers or clinical endpoints, such as evaluation of cancer treatment response, disease diagnosis for autoimmune disorders, and identification of critical quality attributes for tissue and cell engineering. These efforts will support the development of a comprehensive atlas for robust interpretation of label-free images with respect to biological outcomes. Recently, a pan-cancer T cell atlas has been developed based on genomic, pathological, and clinical features from over 350 patients across multiple cohorts to identify T cell stress response as a novel biomarker for immunotherapy resistance.^104^ Such an atlas for label-free microscopy, together with thorough validation, lays the foundation for future applications of label-free microscopy as a stand-alone analytical tool to identify, characterize, and monitor cell or molecular subsets over time and space. Multivariate predictive models based on label-free microscopy features can then inform important decisions in cancer, autoimmune disease, and tissue and cell engineering.

## 5 Conclusion

Label-free microscopy offers non-invasive assessment of complex biological systems that span several key biomedical applications, including but not limited to cancer research, autoimmune disease, and cell and tissue engineering. Current label-free microscopy modalities have multiple benefits over standard assays, allowing non-invasive characterization of cellular morphology, dynamics, and molecular features. Improvements in instrumentation and AI-assisted techniques will enable label-free microscopy with high-speed, resolution, and depth. The use of supervised and unsupervised machine learning and automated image segmentation will further support label-free image analysis and data visualization. Centralization and standardization of the image analysis workflow is also important for data integrity and adoption of label-free microscopy. In addition, data validation with robust biological interpretation will facilitate the translation of label-free microscopy to study heterogeneous biological systems and dynamic cellular processes.

## Data Availability

Data sharing is not applicable to this article, as no new data were created or analyzed.
